# Treatment outcomes of advanced/metastatic extramammary Paget's disease in Korean patients: KCSG‐RC20‐06

**DOI:** 10.1002/cam4.6190

**Published:** 2023-06-01

**Authors:** Byeong Seok Sohn, Jeongeun Kim, Miso Kim, Jung Yong Hong, Jieun Lee, Song Ee Park, Hyojeong Kim, Hyo Jin Lee, Eun Joo Kang, Soon Il Lee, In Hee Lee, Seok Jae Huh, Jeongmin Jo, Ho Young Kim

**Affiliations:** ^1^ Department of Internal Medicine, Sanggye Paik Hospital Inje University College of Medicine Seoul South Korea; ^2^ Department of Oncology, Asan Medical Center University of Ulsan College of Medicine Seoul South Korea; ^3^ Department of Internal Medicine Seoul National University College of Medicine and Seoul National University Hospital Seoul South Korea; ^4^ Division of Hematology‐Oncology, Department of Medicine Samsung Medical Center, Sungkyunkwan University School of Medicine Seoul South Korea; ^5^ Division of Medical Oncology, Department of Internal Medicine Seoul St. Mary's Hospital, College of Medicine, The Catholic University of Korea Seoul South Korea; ^6^ Division of Hematology‐Oncology, Department of Medicine Chung‐Ang University College of Medicine Seoul South Korea; ^7^ Division of Hemato‐Oncology, Department of Internal Medicine Pusan National University School of Medicine Busan South Korea; ^8^ Division of Hematology/Oncology, Department of Internal Medicine College of Medicine, Chungnam National University Daejeon South Korea; ^9^ Division of Hematology‐Oncology, Department of Internal Medicine Korea University Guro Hospital, Korea University College of Medicine Seoul South Korea; ^10^ Division of Hematology‐Oncology, Department of Internal Medicine Dankook University College of Medicine Cheonan South Korea; ^11^ Department of Oncology/Hematology Kyungpook National University Chilgok Hospital, School of Medicine, Kyungpook National University Daegu South Korea; ^12^ Department of Internal Medicine Dong‐A University College of Medicine Busan South Korea; ^13^ Department of Hematology‐Oncology Ewha Womans University Medical Center Seoul South Korea; ^14^ Department of Hematological Oncology Hallym University Sacred Heart Hospital, Hallym University College of Medicine Anyang‐si South Korea

**Keywords:** chemotherapy, metastatic extramammary Paget's disease, overall survival, progression‐free survival, trastuzumab

## Abstract

**Background:**

Extramammary Paget's disease (EMPD) is rare. There are no standard treatments due to its rarity and few clinical trials.

**Methods:**

The objective of this multicenter study was to investigate treatment outcomes of Korean patients with advanced/metastatic EMPD. Data were collected retrospectively from 14 institutions participating in Korean Cancer Study Group (KCSG) Rare Cancer Committee.

**Results:**

A total of 37 patients were identified. Of these 37 patients, 6 received locoregional therapy as a first‐line treatment. In 31 patients who received systemic chemotherapy as a first‐line treatment, platinum‐based chemotherapy (*n* = 22) achieved an objective response rate (ORR) of 45.5% and a median progression‐free survival (PFS) of 7.89 months. Taxane‐based chemotherapy (*n* = 8) achieved an objective response rate of 62.5% and median PFS of 9.73 months. In second‐line chemotherapy, platinum‐based chemotherapy (*n* = 4) had a disease control rate (DCR) of 75.0% and median PFS of 3.45 months. Taxane‐based chemotherapy (*n* = 8) had a DCR of 75.0% and a median PFS of 8.67 months. Six patients received anti‐human epidermal growth factor receptor 2 (HER2) antibody during first‐ and second‐line chemotherapy. Overall, systemic chemotherapy combined with anti‐HER2 antibody had an ORR of 100% and a median PFS of 13.31 months. The ORR and PFS with systemic chemotherapy combined with trastuzumab was better than platinum‐ and taxane‐based chemotherapy only.

**Conclusions:**

Due to its rarity, advanced or metastatic EMPD still has no established standard treatment. Results of our study indicate that the combination of trastuzumab with taxane has longer survival than trastuzumab monotherapy or conventional platinum‐ or taxane‐based chemotherapy.

## Abstract 2

Due to its rarity, advanced or metastatic extramammary Paget's disease (EMPD) still has no established standard treatment and little data on clinical outcomes. In this study, we found that there is no survival difference between platinum‐based chemotherapy and taxane‐based chemotherapy, and trastuzumab with taxane has longer survival than trastuzumab monotherapy or conventional platinum‐ or taxane‐based chemotherapy.

## 1 INTRODUCTION

Extramammary Paget's disease (EMPD) is a rare intraepithelial adenocarcinoma. It is mainly manifested as either a primary cutaneous adenocarcinoma, but it can also be manifested as secondary cutaneous involvement from cancers originating in the urinary tract or lower gastrointestinal tract. The cell of origin for primary cutaneous EMPD still remains controversial. While it is reported that adenocarcinoma originating from underlying apocrine or eccrine glands has spread to the epithelium, it has also been reported as originating from pluripotent stem cells or Toker cells.^1^, ^2^ Also, it is known that the genetic alteration related to tumor development include the mutations in PIK3CA, AKT1, ERBB2, and RAS/RAF pathway.^3^, ^4^ Histopathologically, EMPD is very similar to Paget's disease of the breast. Large malignant epithelial cells are distributed individually or in small clusters among normal epithelial cells, showing variable ductal differentiation and poorly circumscribed proliferation.

EMPD usually presents in the form of eczema‐like plaques with well‐defined boundaries and occurs predominantly in the anogenital region. Occasionally, it manifests as multifocal and bilateral lesions. The vulva is the predominant site of occurrence and, according to report, accounts for up to 65.0% of all cases of EMPD.^5^ However, it accounts for less than 1% of all malignancies originating in the vulvar.^6^ The next common site of occurrence is the perianal region (19.8%) followed by male genitalia such as scrotum and penis (13.7%).^5^ In rare cases, EMPD is observed in the axilla, buttocks, thighs, eyelids, external auditory canal, and other areas rich in apocrine gland.^5^ The incidence of EMPD in men and women seems to be reversed between East and West.^7^, ^8^ In East, EMPD is more common in males and the male‐to‐female ratio is reported to be 3.5:1–3.9:1. Whereas in the West, it is more prevalent in females and the male‐to‐female ratio is reported to be 1:2–1:7.^9^ The majority of EMPD patients are diagnosed in their 60s and 70s.^8^, ^9^, ^10^ However, the peak age at which it occurs varies depending on the locations of its occurrence. When it occurs in the female genitalia, it is observed mainly between the age of 50 and 65, and in the scrotum and penis, it is mainly observed in their 70s.^9^, ^10^

Most patients with EMPD are diagnosed and treated in the stage of carcinoma in situ.^11^ The prognosis of these patients is usually good. However, once EMPD begins to invade into the dermis and becomes invasive EMPD, it acquires the ability to metastasize remotely, causing lymph node (LN) metastasis even in patients with microscopic dermal invasion.^12^ Over one‐third of patients who develop LN metastasis eventually have distant metastases.^13^

The prognosis for patients with distant metastases is very poor.^14^ Among several chemotherapeutic regimens, low‐dose 5‐fluorouracil/cisplatin and docetaxel monotherapy have been commonly used to treat advanced/metastatic EMPD.^15^, ^16^ The overexpression of Human epidermal growth factor receptor 2 (HER2) in the tumor of some patients with EMPD suggests that concomitant use of trastuzumab may be beneficial for treatment.^17^ However, patients with metastatic EMPD are known to have a median OS of 1.5 years and a 5‐year survival rate of 7%.^13^ Hence, there is still an urgent need for new therapies to treat metastatic EMPD.

Nevertheless, there is still no established standard treatment for advanced or metastatic EMPD. Studies on real‐world results of current practice are also rare. In this regard, we planned a multicenter retrospective study to investigate current treatment patterns and compare clinical outcomes of Korean advanced or metastatic EMPD patients.

## 2 PATIENTS AND METHODS

### 2.1 Patients

A total of 14 medical center affiliated with Rare Cancer Committee of Korean Cancer Study Group (KCSG) participated in this study. As a retrospective study, medical records of 14 participating centers were searched from January 2004 to December 2018. Fifty‐three patients with advanced or metastatic EMPD was found. Ten patients who refused treatment after initial diagnosis or did not receive any kind of treatment for his or her recurrent disease were excluded. Six patients who had secondary EMPD were also excluded. Four cases were related to rectal cancer. One case was related to bile tract cancer and one case was related to ureter cancer. Clinical characteristics were retrospectively collected from medical records including age, gender, primary site, relapse/metastatic sites, HER2 status, laboratory data, the type of treatments, treatment regimens, the start & end date of treatment, outcomes of treatment, and survivals. The study protocol was approved by the Institutional Review Board of each participating hospital.

### 2.2 Methods

Responses to treatment were determined by investigators at each participating institution according to RECIST criteria. Scheduled follow‐up with computed tomography was performed every 3 or 4 cycles of chemotherapy or whenever clinically indicated in accordance with policies of participating centers. Objective response rate (ORR) was determined by the percent of patients who achieved a complete response or partial response to the treatment. Progression‐free survival (PFS) was calculated from the date of the initiation of treatment to the date of disease progression or death from any cause. Overall survival (OS) was calculated from the date of the initiation of first‐line treatment for recurrence or metastasis to the date of death from any cause.

### 2.3 Statistical analysis

Descriptive statistics was used to analyze clinical data. The association between clinical characteristics and response to chemotherapy was determined using Fisher's exact test. PFS and OS were analyzed using the Kaplan–Meier method and compared with the log‐rank test. Statistical significance was defined by *p* < 0.05. All data were analyzed with the Statistical Package for Social Sciences 24.0 (IBM Corp.) and MedCalc 20.014 (MedCalc Software).

## 3 RESULTS

### 3.1 Patient characteristics

Clinical characteristics of the 37 patients with primary EMPD are summarized in Table 1. The median age of these patients was 68 years (range: 47–80 years). The male: female ratio was 5.17. The most prevalent site of primary was the urogenital region (including scrotum and vulva) in 29 of 37 (78.4%) patients. Four patients had locally advanced, unresectable disease. Twenty‐one patients had relapsed disease and 12 patients initially had metastatic disease. LN metastasis was detected in 35 (94.6%) of 37 patients. Distant metastasis was detected in 17 (45.9%) of 37 patients.

**TABLE 1:** Patient's characteristics.

Characteristics	*N*. of patients (%)
Age	
(Years, median)	67.95 (47–80)
Gender	
Male	31 (83.8)
Female	6 (16.2)
ECOG PS	
0	3 (8.1)
1	18 (48.6)
2	4 (10.8)
3	1 (2.7)
Unknown	11 (29.7)
Primary site	
Urogenital^a^	29 (78.4)
Groins	3 (8.1)
Genital+Perianal	2 (5.4)
Perianal	1 (2.7)
Others^b^	2 (5.4)
Disease status	
Locally advanced	4 (10.8)
Initially metastatic	12 (32.4)
Recurred	21 (46.8)
Lymph node involvement (*N* = 35)	
Inguinal	33 (94.3)
Pelvic	24 (68.6)
Distant	20 (57.1)
Distant metastasis (*N* = 17)	
Bone	9 (52.9)
Lung	8 (47.1)
Liver	4 (23.5)
Others	5 (29.4)^c^
HER‐2 positivity (*N* = 22)	
Negative	10 (45.5)
Positive	12 (54.5)

^a^ Vulva and scrotum.

^b^ Axillar and esophagus.

^c^ One bone marrow, one brain, one pleura, one skin, and one spleen metastasis.

### 3.2 Clinical outcomes

In 37 patients, three patients received palliative radiation therapy only, three patients received locoregional therapy, and 31 patients received systemic chemotherapy (Appendix 1). In the three patients receiving palliative radiation therapy only, the OS of one patient receiving radiation therapy for brain metastasis was 2.4 months. For another patient with bone metastasis, the OS was 3.1 months until loss to follow‐up. For another patient with lung metastasis, the OS was 10.3 months.

In three of 37 patients receiving locoregional therapy, one patient who had advanced scrotal EMPD with multiple inguinal LN metastases received wide excision followed by radiation therapy. This patient had PFS and OS of 90.5 months until loss to follow‐up. Another patient who had recurred EMPD at axillary LN received excision followed by radiation therapy had PFS and OS of 5.7 months until loss to follow‐up. The other patient who had scrotal EMPD with right inguinal LN metastases and received wide excision followed by concurrent chemoradiation therapy (5400 cGy, trastuzumab+pertuzumab+cisplatin+capecitabine) had of PFS and OS of 58.4 months. The patient was alive at the time of manuscript was written.

**FIGURE 1:**
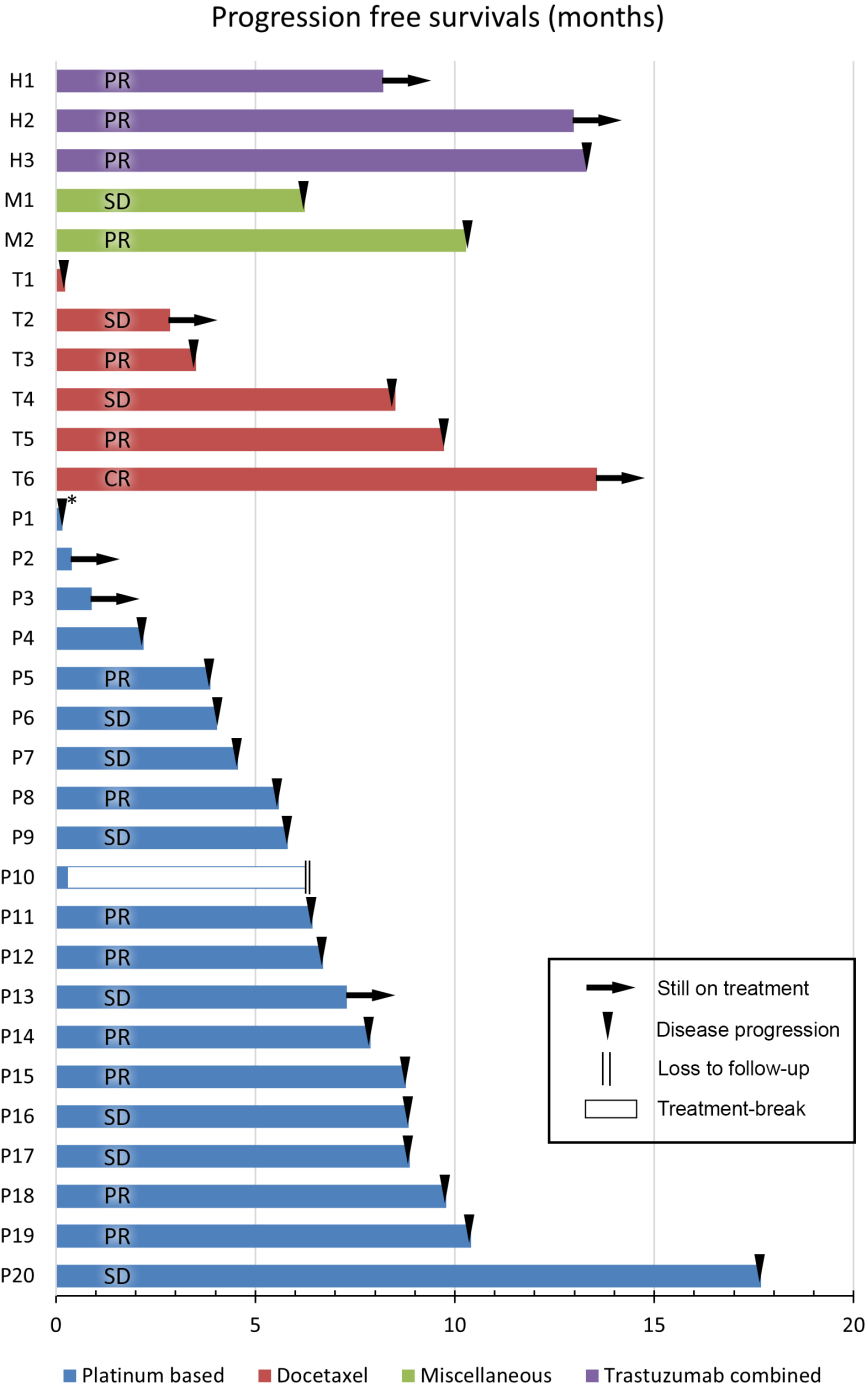
Outcomes of first‐line systemic chemotherapy.

**FIGURE 2:**
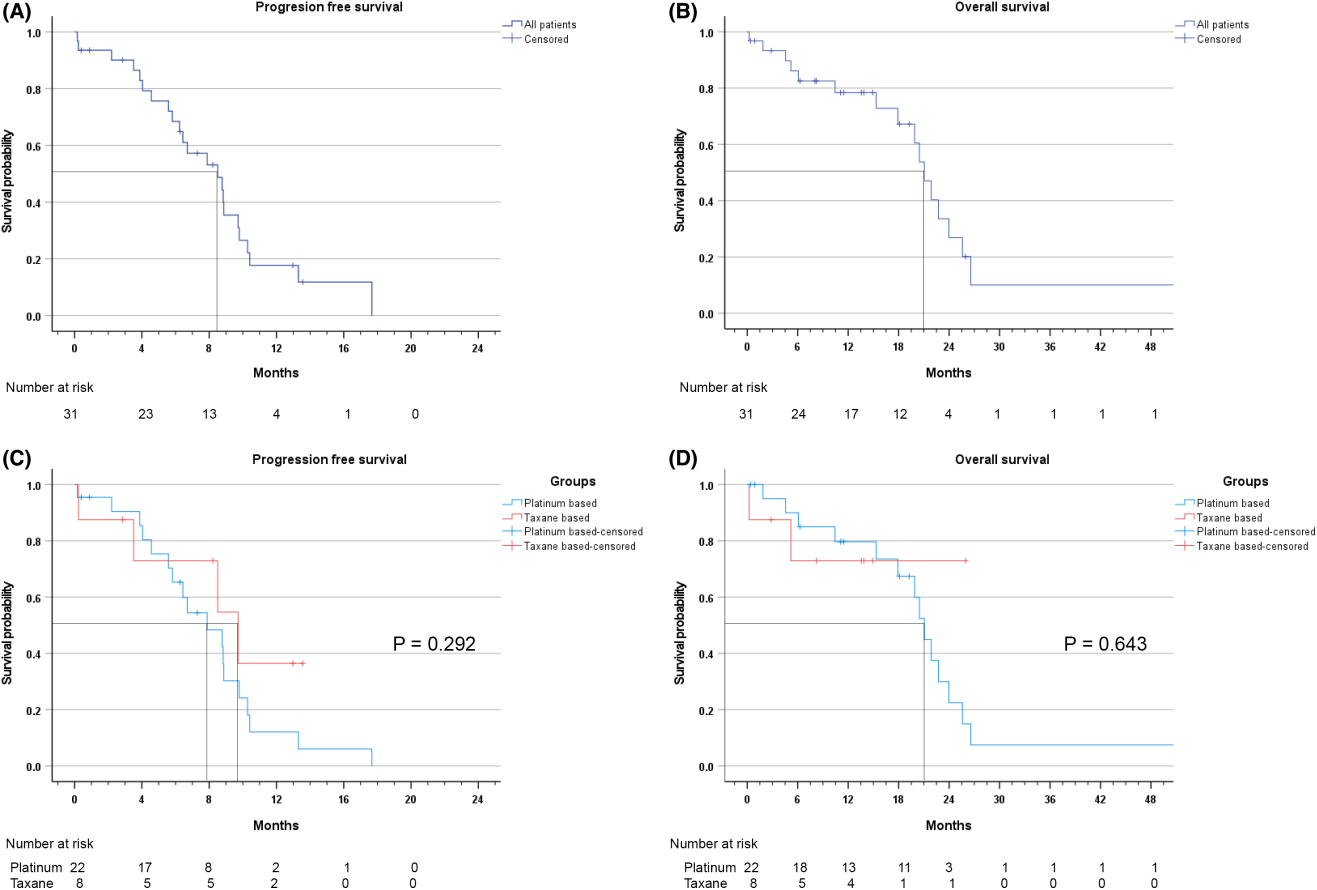
Comparison of survivals according to chemotherapy regimen. (A) Progression‐free survival of first‐line chemotherapy, (B) overall survival from first‐line chemotherapy, (C) progression‐free survival of first‐line chemotherapy according to chemotherapy group (platinum based vs. taxane based), (D) overall survival from first‐line chemotherapy according to chemotherapy group (platinum based vs. taxane based).

Thirty‐one of 37 patients initially received systemic chemotherapy as a first‐line treatment. The overall ORR was 48.4% (15 of 31). The median PFS was 8.51 months (95% confidence interval [CI]: 5.45–11.57). The median OS was 21.06 months (95% CI: 18.75–23.37). In 31 patients who were initially treated with systemic chemotherapy (Figure 1), 22 patients received platinum‐based chemotherapy and 8 patients received taxane‐based chemotherapy as a first‐line chemotherapy. There were no statistically significant differences between two groups in age, gender, ECOG PS, primary sites, disease status, LN station, metastatic sites or HER2 positivity (Appendix 2). As a first‐line chemotherapy, patients who received platinum‐based chemotherapy achieved an ORR of 45.5% (10 of 22), a disease control rate (DCR) of 72.7% (16 of 22), a median PFS of 7.89 months (95% CI: 4.87–10.90), and a median OS of 21.06 months (95% CI: 10.10–23.02). Also, the patient who received taxane‐based chemotherapy had an ORR of 62.5% (5 of 8), a DCR of 87.5% (7 of 8), and a median PFS of 9.73 months (95% CI: 3.05–16.40). During a median follow‐up time of 13.9 months (range: 0.23–25.95) for 8 patients, median OS was not yet reached. There was no significant difference in PFS or OS between platinum‐based chemotherapy and taxane‐based chemotherapy (*p* = 0.292 and *p* = 0.643, respectively) (Table 2). There was no statistically significant difference in PFS or OS between 5‐fluorouracil/cisplatin and docetaxel monotherapy either (*p* = 0.637 and *p* = 0.702, respectively). The remaining one patient received a combination of platinum and taxane. The patient received carboplatin+docetaxel chemotherapy, and he had a stable disease with a PFS of 6.24 months and an OS of 8.05 months until loss to follow‐up. Overall PFS and OS of those treated with first‐line chemotherapy are shown in Figure 2.

**TABLE 2:** Clinical outcomes of platinum‐ or taxane‐based regimen.

	Platinum‐based	Taxane‐based	*p*‐value
First‐line therapy			
Progression‐free survival	7.89 months (95% CI: 4.87–10.90)	9.73 months (95% CI: 3.05–16.40)	0.292
Overall survival	21.06 months (95% CI: 10.10–23.02)	13.9 months (range: 0.23–25.95)	0.643
Second‐line therapy			
Progression‐free survival	3.45 months (95% CI: 0.30–6.60)	8.67 months (95% CI: 3.68–13.67)	0.525
Overall survival	13.78 months (95% CI: –)	13.37 months (95% CI: 0–26.93)	0.922

**FIGURE 3:**
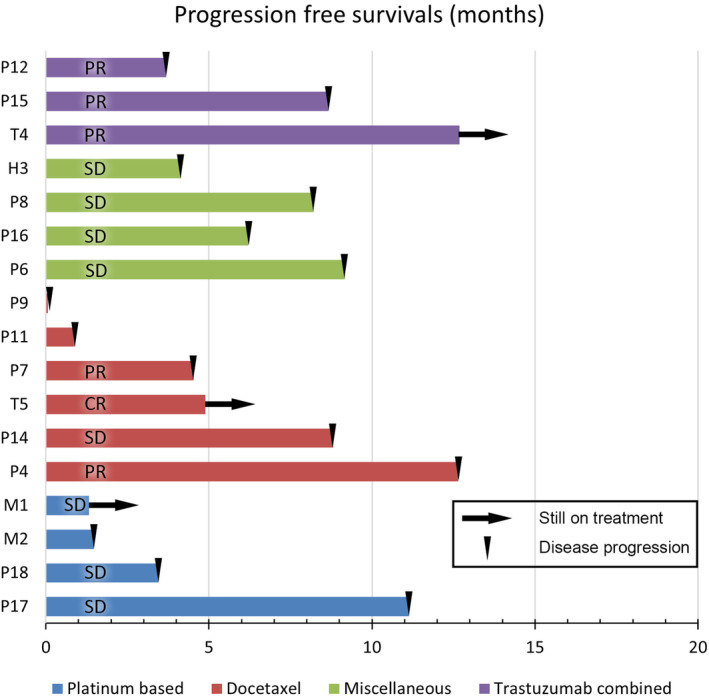
Outcomes of second line of chemotherapy.

Twenty‐five patients experienced disease progression during first‐line chemotherapy. Seventeen of 25 patients had received second‐line chemotherapy (Figure 3). The overall ORR was 35.3% (6 of 17) and median PFS was 6.21 months (95% CI: 0–12.66). In second‐line chemotherapy, platinum‐based chemotherapy was used in four patients and taxane‐based chemotherapy was used in eight patients. At the second‐line chemotherapy, patients who received platinum‐based chemotherapy had no objective response, but had a DCR of 75.0% (3 of 4) with a median PFS of 3.45 months (95% CI: 0.30–6.60). Patients who received taxane‐based chemotherapy achieved an ORR of 62.5% (5 of 8), a DCR of 75.0% (6 of 8), and a median PFS of 8.67 months (95% CI: 3.68–13.67). In the remaining five patients who received second‐line chemotherapy, two patients who received capecitabine each acquired SD, with PFS of 6.21 months and 9.17 months, respectively. The patient who received carboplatin+paclitaxel at second‐line chemotherapy had SD and a PFS of 4.14 months. The patient who received cyclophosphamide+doxorubicin+vincristine chemotherapy (CAV) achieved SD and a PFS of 8.21 months. The patient who received gemcitabine+trastuzumab biosimilar (samfenet®) achieved PR but a PFS of 3.68 months.

Fourteen patients experienced disease progression during second‐line chemotherapy. Six of 14 patients received third‐line chemotherapy. The patient who received cisplatin+dacarbazine chemotherapy had PD at best response and a PFS of 0.23 months. The patient who received methotrexate+vinblastine+doxorubicin+cisplatin (M‐VAC) achieved PR and a PFS of 4.93 months. Of two patients who received docetaxel monotherapy, one patient had PD and a PFS of 1.84 months, and the other patient had SD and a PFS of 6.74 months. The patient who received etoposide monotherapy achieved PR and a PFS of 2.99 months. The patient who received doxorubicin monotherapy achieved SD and a PFS of 6.97 months. Six patients experienced disease progression during third‐line chemotherapy. Three of these six patients received fourth‐line chemotherapy (cyclophosphamide+etoposide+vincristine+doxorubicin, trastuzumab monotherapy, or 5‐fluorouracil/cisplatin regimen was used in each patient) and all initially had PD at best response with PFS of 2.99, 1.84, and 0.26 months, respectively. One of the three patients received fifth‐line chemotherapy with etoposide after trastuzumab monotherapy. The patient had PD at best response and a PFS of 1.41 months. Summarizing the treatment results of third‐line, fourth‐line, and fifth‐line chemotherapy, there were objective responses in 3 out of 10 treatments, and the median PFS of 10 treatment was 2.42 months.

Systemic chemotherapy combined with anti‐HER2 antibody (trastuzumab or trastuzumab biosimilar) was used in six patients with HER2‐positive EMPD: three in first‐line, three in second‐line, and one in fourth‐line (Table 3). In six patients who received anti‐HER2 antibody during first‐ and second‐line chemotherapy, systemic chemotherapy combined with anti‐HER2 antibody had an ORR of 100% and a median PFS of 13.31 months. One patient who received trastuzumab monotherapy at fourth‐line had PD at best response and a PFS of 1.84 months.

**TABLE 3:** Clinical outcomes of primary EMPD with anti‐HER2 antibodies.

	Best overall response	Progression‐free survival	Overall survival^b^
First‐line therapy			
Cisplatin+capecitabine+trastuzumab	PR	13.31	20.83
Paclitaxel+trastuzumab	PR	12.98^c^	13.86^c^
Paclitaxel+trastuzumab	PR	8.21^c^	8.67^c^
Second‐line therapy			
Paclitaxel+trastuzumab	PR	12.68^c^	12.87^c^
Paclitaxel+trastuzumab	PR	8.67	8.80^c^
Gemcitabine+trastuzumab biosimilar^a^	PR	3.68	4.63^c^
Fourth line therapy			
Trastuzumab	PD	1.84	4.53

^a^ Samfenet®.

^b^ From the start of trastuzumab based chemotherapy.

^c^ Patient was on treatment and alive at the end of the study.

In 31 patients treated with first‐line systemic chemotherapy, treatment response was not associated with clinical factors including age, performance status (PS), primary sites, disease status, LN station, the sites of metastasis, HER2 positivity, the type of treatment (platinum vs. taxane), and CK7/CK20/CEA positivity (Appendix 3). Progression‐free survival (PFS) was associated with PS (0,1 vs. 2,3). Good performance status (PS: 0,1) had longer PFS than poor performance status (PS: 2,3) (*N* = 19 vs. 4, 8.87 [95% CI: 7.40–10.34] vs. 3.88 [95% CI: 0–8.16], *p* = 0.032). In first‐line chemotherapy, three patients who received chemotherapy combined with trastuzumab had numerically longer PFS than those received platinum‐ and taxane‐based chemotherapy (*N* = 3 vs. 27, 13.31 months vs. 7.89 months, *p* = 0.080). In 12 patients who had HER2‐positive EMPD, nine patients received first‐line chemotherapy. In these nine patients, patients who received chemotherapy combined with trastuzumab had longer PFS than those received platinum‐ and taxane‐based chemotherapy (*N* = 3 vs. 6, 13.31 months vs. 6.70 months, *p* = 0.024). Of the 31 patients receiving chemotherapy, none received radiotherapy or surgery other than palliative purposes that could affect the determination of response during chemotherapy. Outcomes of serial systemic chemotherapy in primary EMPD in 31 patients showed in Appendix 4.

## 4 DISCUSSION

In this study, a male predominance was observed in advanced/metastatic disease in Korean patients. In patients who received systemic chemotherapy as a first‐line therapy, the median PFS was 8.51 months and the OS was 21.06 months. Patients who received platinum‐based chemotherapy at first‐line chemotherapy had a median PFS of 7.89 months and a median OS of 21.06 months. The patient who received taxane‐based chemotherapy at first‐line chemotherapy had a median PFS of 9.73 months. The median OS was not reached at 13.9 months of median follow‐up time. Above all, we found that patients receiving systemic chemotherapy combined with trastuzumab had longer PFS than patients receiving conventional chemotherapy only. Also, we found that a significant number of patients did not receive more than a second line of chemotherapy.

EMPD tends to be more common in males in Asians. Cheng et al. have reported a 3.5:1 of (389 vs. 110) male predominance in Taiwan patients.^7^ Lee et al. have reported a 3.9:1 (154 vs. 40) in Korean patients.^8^ Hatta et al. have reported a male to female ratio of 2.52:1 (55 vs. 21) in Japanese patients.^11^ The male‐to‐female ratio in our study was 5.17:1, somewhat higher than that of previous studies. It was once postulated that the cultural difference–conservative tendency could make the incidence of EMPD in Asian females to be underestimated.^7^ However, the cultural difference or conservative tendency is unlikely that patients with advanced or metastatic disease will be reluctant to seek medical service, resulting in reversed sex ratio. Therefore, to define the reason for the reverse ratio in male‐to‐female between races, further studies on the pathogenesis of EMPD are necessary.

In our study, platinum‐based chemotherapy was preferred to docetaxel‐based chemotherapy as first‐line treatment. More than a half of patient (22 of 31) received platinum‐based chemotherapy for first‐line chemotherapy. Taxane‐based chemotherapy was used in eight patients for first‐line treatment. Because taxanes are not covered by Korean Health Insurance, clinicians might have preferred platinum‐based chemotherapy as their first‐line prescription. Numerically, the PFS of taxane‐based chemotherapy (docetaxel monotherapy) seems to be better than that of platinum‐based chemotherapy (FP). However, taxane‐ or platinum‐based chemotherapy had no statistical difference in survival as a first‐line treatment. Because our comparison included only eight patients treated with taxane‐based chemotherapy, the numerical difference should be re‐evaluated with a larger number of patients. Although these two regimens are the most commonly used in advanced/metastatic EMPD, only 5 of 19 patients who received FP as a first‐line chemotherapy received docetaxel monotherapy for second‐line chemotherapy. None of the eight patients who received docetaxel monotherapy as a first‐line chemotherapy received platinum‐based chemotherapy for second‐line chemotherapy. Therefore, in this study, it is not possible to determine which chemotherapy is better as first‐line chemotherapy.

Our results of platinum‐ and taxane‐based chemotherapy are comparable to those of previous studies. Kariya et al. have reported a beneficial effect of low‐dose 5‐fluorouracil/cisplatin regimen in one patient with metastatic EMPD.^18^ Tokuda et al. have reported an ORR of 58.8% (10 of 17), a median duration of response of 5 months (range: 1.5–24 months), and a median OS of 12 months (range: 5–51 months).^15^ Kato et al. have reported an ORR of 50%, a median PFS of 25.0 weeks, and a median OS of 77.4 weeks with low‐dose 5‐fluorouracil/cisplatin therapy.^19^ Most of our patients receiving platinum‐based chemotherapy were treated with conventional dose of 5‐fluorouracil/cisplatin. Although numerically our median PFS of 7.89 months at first‐line treatment seems better than that of previous low‐dose 5‐fluorouracil/cisplatin by 2 months. It is not clear whether conventional dose 5‐fluorourail/cisplatin therapy would be more efficient than low does 5‐fluorourail/cisplatin therapy. Yoshino et al. have reported an ORR of 58% with a PFS of 7.1 months and an OS of 16.6 months with docetaxel as the first‐line treatment.^16^ Our patients receiving docetaxel as a first‐line treatment had a median PFS of 8.51 months with a median OS not reaching during 13.9 months of median follow‐up time.

Our data demonstrate that most patients underwent median 2 treatment‐line (range: 1–5; second line in 17 of 25 patients; third line in 6 of 14; fourth line in 3 of 6; and fifth line in 1 of 3). Most patients received only first or second line of chemotherapy. There was no standard regimen after second line of chemotherapy. After second line of chemotherapy, the response was also poor (response rate: 20% [2 of 10], median PFS <3 months). Considering that about half of patients just underwent one or two lines of chemotherapy and that a few people receive third or more line chemotherapy, to improve patient’ survival, we should focus on developing more efficacious regimen and its application on early line of treatment before patient's deterioration.

Histologically, EMPD has very similar characteristics to breast Paget's disease. In breast Paget's disease, HER2 tends to be amplified or overexpressed in most patients.^20^ Similarly, 15%–60% of patients with EMPD have HER2 protein overexpression and gene amplification.^20^, ^21^, ^22^ Based on results of this study, clinical studies targeting HER2 have reported the results of trastuzumab monotherapy in patients with metastatic EMPD. The PFS of these studies ranged from 6–12 months.^23^, ^24^ In recent case reports, the combination therapy of trastuzumab and paclitaxel was effective in patients with metastatic EMPD.^25^, ^26^, ^27^

Contrary to results of previous study with trastuzumab monotherapy,^23^, ^24^ trastuzumab alone in our study is not meaningful as a fourth‐line treatment with a PFS of 1.84 months. Also, trastuzumab combined with gemcitabine had a PFS of 3.68 months. It is unclear whether it is because it is the later line (fourth line) therapy, the limitation of monotherapy, or problem in its combination with gemcitabine. A few case reports have tried gemcitabine monotherapy or combination therapy in metastatic EMPD and found that the efficacy is modest at best.^28^, ^29^, ^30^ Therefore, if anti‐HER2 antibody treatment should be attempted as a monotherapy, trastuzumab emtansine might be another option.^31^ Results of our study indicate that the combination of trastuzumab with other anti‐cancer drugs has longer survival than trastuzumab monotherapy or conventional platinum‐ or taxane‐based chemotherapy. Considering the efficacy of the paclitaxel and trastuzumab combination chemotherapy in our study and the difficulty for most patients to receive third‐ or fourth‐line chemotherapy, trastuzumab combined with systemic chemotherapy—showing a median PFS of 13.31 months—could be the first‐line chemotherapy.

We performed a statistical analysis to compare clinical factors and clinical outcomes, but most of the data had many missing values, making it difficult to properly process statistics. Therefore, it should be considered that some of our statistical results have biases and limitations in interpretation. In this study, no specific clinical factor influenced the clinical outcomes except the well‐known prognosticator—PS. However, due to small sized and the nature of the retrospective study, this result needs to be confirmed through another large sized study. Several other cytotoxic chemotherapeutic regimens were tried at various lines of treatment. However, outcomes of these regimens were not superior to those of platinum‐ or taxane‐based chemotherapy used in first‐line or second‐line treatment. Therefore, there is no reason to prefer these regimens to platinum‐ or taxane‐based chemotherapy without compelling indications. Recently, pyrotinib, a tyrosine kinase inhibitor, have been attempted in patients harboring HER2 mutations. Immune checkpoint inhibitors in PD‐L1 positive cancer are being attempted. They might be helpful in the treatment of metastatic EMPD.^32^, ^33^

In summary, platinum‐ and taxane‐based chemotherapy showed similar efficacy in advanced/metastatic EMPD. However, there was no further improvement in treatment or clinical outcomes with conventional cytotoxic chemotherapy so far. To improve the patient's outcomes, it is urgent to introduce new treatment strategy such as targeted therapies and immune checkpoint therapies. The combination of anti‐HER2 antibody could be a turning point in the treatment for patients with advanced/metastatic EMPD. To improve the patient's outcomes, effort to introduce new treatments is mandatory and its application on early line of treatment before patient's deterioration.

## AUTHOR CONTRIBUTIONS

**Byeong Seok Sohn:** Conceptualization (equal); data curation (equal); formal analysis (equal); resources (equal); writing – original draft (lead). **Jeongeun Kim:** Data curation (equal); writing – review and editing (equal). **Miso Kim:** Data curation (equal); writing – review and editing (equal). **Jung Yong Hong:** Data curation (equal); writing – review and editing (equal). **Jieun Lee:** Data curation (equal); writing – review and editing (equal). **Song Ee Park:** Writing – review and editing (equal). **Hyojeong Kim:** Data curation (equal); writing – review and editing (equal). **Hyo Jin Lee:** Data curation (equal); writing – review and editing (equal). **Eun Joo Kang:** Data curation (equal); writing – review and editing (equal). **Soon Il Lee:** Data curation (equal); writing – review and editing (equal). **In Hee Lee:** Data curation (equal); writing – review and editing (equal). **Seok Jae Huh:** Data curation (equal); writing – review and editing (equal). **Jeongmin Jo:** Data curation (equal); writing – review and editing (equal). **Ho Young Kim:** Data curation (equal); writing – review and editing (equal).

## CONFLICT OF INTEREST STATEMENT

All authors declare no conflict of interests.

## Data Availability

Data available in article supplementary material.

## APPENDIX 1 Individual Information chart for 34 patients

**Table:**

	ID	Gender	Age	Follow‐up time (months)	Disease status	Initial metastatic sites	Treatment	First‐line treatment	Cycles	Best overall response	PFS	Second‐line treatment	Cycles	Best overall response	PFS	OS
1	H1	M	57	9	Initially metastatic	R.LNs, bone, liver, spleen	CTx	Paclitaxel 80 mg/m^2^ weekly, 6 weeks on & 2 weeks off, every 8 weeks; trastuzumab 4 mg/kg on week 1, then 2 mg/kg starting week 2, weekly		PR	8.21^a^					8.21^b^
2	H2	M	61	15	Recurred	R.LNs, D.LNs, bone	CTx	Paclitaxel 80 mg/m^2^ weekly, 6 weeks on & 2 weeks off, every 8 weeks; trastuzumab 4 mg/kg on week 1, then 2 mg/kg starting week 2, weekly	11	PR	12.98^a^					13.86^b^
3	H3	M	59	21	Initially metastatic	R.LNs, P.LNs, D.LNs	CTx	Cisplatin 60 mg/m^2^ D1, capecitabine 1000 mg/m^2^ BD D1‐14, trastuzumab 8 mg/kg on the first cycle, then 6 mg/kg starting with the second cycle, every 3 weeks	3	PR	13.31	Paclitaxel 175 mg/m^2^, Carboplatin 5AUC	3	SD	8.80	29.70
4	M1	M	69	9	Recurred	R.LNs, P.LNs, D.LNs, bone	CTx	Carboplatin 2.5AUC D1, docetaxel 37.5 mg/m^2^ D1, every 3 weeks	7	SD	6.24	Cisplatin 60 mg/m^2^ D1, 5‐FU 1000 mg/m^2^ D1‐4, every 4 weeks	1	SD	1.31^a^	8.05^b^
5	M2	M	58	23	Initially metastatic	R.LNs, P.LNs, D.LNs, lung	CTx	Cisplatin 60 mg/m^2^ D1, etoposide 50 mg/m^2^ PO BD D1‐3, every 3 weeks	11	PR	10.28	Cisplatin 60 mg/m^2^ D1, 5‐FU 1000 mg/m^2^ D1‐4, every 4 weeks	2	PD	1.48	15.34
6	P1	M	60	2	Recurred	R.LNs, liver, lung	CTx	Cisplatin 60 mg/m^2^ D1, 5‐FU 1000 mg/m^2^ D1‐4, every 4 weeks	2	PD	0.16					1.87
7	P2	M	48	2	Recurred	R.LNs, P.LNs, D.LNs	CTx	Cisplatin 60 mg/m^2^ D1, 5‐FU 1000 mg/m^2^ D1‐4, every 4 weeks	1	NE	0.39^a^					0.39^b^
8	P3	M	60	9	Initially metastatic	R.LNs, P.LNs, D.LNs	CTx	Cisplatin 60 mg/m^2^ D1, 5‐FU 1000 mg/m^2^ D1‐5, every 4 weeks	2	NE	0.89^a^					0.89^b^
9	P4	F	72	24	Initially metastatic	R.LNs, P.LNs, D.LNs	CTx	Cisplatin 75 mg/m^2^ D1, 5‐FU 1000 mg/m^2^ D1‐4, every 4 weeks	2	PD	2.20	Docetaxel 75 mg/m^2^, every 3 weeks	6	PR	12.65	23.98
10	P5	M	69	51	Unresectable	R.LNs	CTx	Cisplatin 60 mg/m^2^ D1, 5‐FU 1200 mg/m^2^ D1‐4, every 4 weeks		PR	3.88					4.57
11	P6	M	76	26	Recurred	R.LNs, P.LNs, D.LNs, lung, pleura	CTx	Cisplatin 50 mg/m^2^ D1, 5‐FU 1000 mg/m^2^ D1‐3, every 4 weeks	6	SD	4.04	Capecitabine 1250 mg/m^2^ bid D1‐14, every 3 weeks	12	SD	9.17	25.59
12	P7	M	58	17	Recurred	R.LNs, P.LNs, D.LNs, lung, liver	CTx	Cisplatin 60 mg/m^2^ D1, 5‐FU 1000 mg/m^2^ D1‐4, every 4 weeks	4	SD	4.57	Docetaxel 75 mg/m^2^, every 3 weeks	5	PR	4.53	11.47^b^
13	P8	F	47	27	Recurred	R.LNs, P.LNs	CTx	Cisplatin 60 mg/m^2^, 5‐FU 1200 mg/m^2^, every 4 weeks	3	PR	5.59	Cyclophosphamide 750 mg/m^2^ D1, doxrubine 37 mg/m^2^ D1, vincristine 2 mg/m^2^ D1, every 3 weeks	6	SD	8.21	26.58
14	P9	M	62	6	Initially metastatic	R.LNs, P.LNs, D.LNs, bone, BM	CTx	Cisplatin 60 mg/m^2^, 5‐FU 1200 mg/m^2^, every 4 weeks	4	SD	5.82	Docetaxel 25 mg/m^2^, weekly	1	PD	0.07	6.08
15	P10	M	72	7	Recurred	R.LNs, P.LNs, D.LNs	CTx	Cisplatin 60 mg/m^2^ D1, 5‐FU 1000 mg/m^2^ D1‐4, every 4 weeks	1	NE	6.28^a^					6.28^b^
16	P11	M	60	11	Recurred	R.LNs, D.LNs, Liver	CTx	Cisplatin 60 mg/m^2^, 5‐FU 1200 mg/m^2^, every 4 weeks	6	PR	6.44	Docetaxel 50 mg/m^2^, every 3 weeks	1	PD	0.89	10.45
17	P12	M	74	14	Recurred	P.LNs, D.LNs, Lung, Bone	CTx	Cisplatin 60 mg/m^2^ D1, 5‐FU 1000 mg/m^2^ D1‐4, every 4 weeks	2	PR	6.70	Gemcitabine 1000 mg/m^2^ D1, D8, sanfenet 8 mg/kg on the first cycle, then 6 mg/kg starting with the second cycle, every 3 weeks	6	PR	3.68	11.10^b^
18	P13	F	62	9	Unresectable	R.LNs	CTx	Cisplatin 60 mg/m^2^ D1, TS‐1 (tegafur/Oteracil/gimeracil) 100 mg/day D1‐14, every 3 weeks	5	NE	7.29^a^					57.23^b^
19	P14	M	70	22	Recurred	R.LNs, bone	CTx	Cisplatin 60 mg/m^2^ D1, 5‐FU 1000 mg/m^2^ D1‐4, every 4 weeks	6	PR	7.89	Docetaxel 60 mg/m^2^, every 3 weeks	6	SD	8.80	21.88
20	P15	F	77	22	Initially metastatic	R.LNs, P.LNs, D.LNs	CTx	Cisplatin 10 mg/m^2^ D1‐5, 5‐FU 600 mg/m^2^ D1‐5	9	PR	8.77	Paclitaxel 80 mg/m^2^ weekly, 6 weeks on & 2 weeks off, every 8 weeks; Trastuzumab 4 mg/kg on week 1, then 2 mg/kg starting week 2, weekly	4	PR	8.67	18.10^b^
21	P16	M	64	21	Recurred	R.LNs, P.LNs, D.LNs	CTx	Cisplatin 60 mg/m^2^ D1, 5‐FU 1000 mg/m^2^ D1‐4, every 4 weeks	6	SD	8.84	Capecitabine 1250 mg/m^2^ bid D1‐14, every 3 weeks	6	SD	6.21	20.47
22	P17	M	55	23	Recurred	R.LNs, P.LNs	CTx	Cisplatin 60 mg/m^2^ D1, 5‐FU 1000 mg/m^2^ D1‐4, every 4 weeks		SD	8.87	Methotrexate 30 mg/m^2^ D1, D15, D22, cisplatin 70 mg/m^2^ D2, vinblastine 3 mg/m^2^ D1, D15, D22, doxorubicin 30 mg/m^2^ D2, every 4 weeks		SD	11.14	22.74
23	P18	M	51	20	Recurred	R.LNs, lung	CTx	Cisplatin 60 mg/m^2^ D1, 5‐FU 1000 mg/m^2^ D1‐4, every 4 weeks	6	PR	9.79	Cisplatin, cyclophosphamide	6	SD	3.45	19.25^b^
24	P19	M	69	24	Initially metastatic	R.LNs, P.LNs	CTx	Cisplatin 55 mg/m^2^ D1, 5‐FU 800 mg/m^2^ D1‐4, every 4 weeks	6	PR	10.41					21.06
25	P20	F	72	19	Initially metastatic	R.LNs, P.LNs, D.LNs	CTx	Cisplatin 60 mg/m^2^, 5‐FU 1200 mg/m^2^, every 4 weeks	3	SD	17.68					17.91
26	T1	M	71	16	Recurred	R.LNs	CTx	Docetaxel 75 mg/m^2^ D1, every 3 weeks		NE	0.23					0.23
27	T2	M	71	6	Initially metastatic	R.LNs, P.LNs, D.LNs	CTx	Docetaxel 75 mg/m^2^, every 3 weeks	5	SD	2.86^a^					2.86^b^
28	T3	F	72	5	Recurred	R.LNs, P.LNs, D.LNs, Lung	CTx	Docetaxel 75 mg/m^2^, every 3 weeks	5	PR	3.52					5.19
29	T4	M	68	27	Initially metastatic	R.LNs, P.LNs, D.LNs	CTx	Docetaxel 60 mg/m^2^, every 3 weeks	4	SD	8.51	Paclitaxel 80 mg/m^2^ weekly, 6 weeks on & 2 weeks off, every 8 weeks; trastuzumab 4 mg/kg on week 1, then 2 mg/kg starting week 2, weekly	6	PR	12.68^a^	25.95^b^
30	T5	M	75	15	Recurred	R.LNs, P.LNs, bone, skin	CTx	Docetaxel 75 mg/m^2^, every 3 weeks	6	PR	9.72	Docetaxel 75 mg/m^2^, every 3 weeks	5	CR	4.90^a^	14.92^b^
31	T6	M	63	22	Recurred	R.LNs, P.LNs, D.LNs	CTx	Docetaxel 75 mg/m^2^ D1, every 3 weeks, 75% DR after #2	6	CR	13.57^a^					13.57^b^
32		M	73	3	Initially metastatic	R.LNs, P.LNs, bone	RT only			NE	1.41^a^					3.13^b^
33		M	58	59	Advanced	R.LNs	Locoregional			NED	58.35^a^					58.35^b^
34		M	67	3	Recurred	Brain, bone	RT only			NE	2.37^a^					2.37
35		M	69	10	Recurred	Lung	RT only			NE	5.55					10.27^b^
36		M	80	7	Recurred	R.LNs	Locoregional			NED	5.72^a^					5.72^b^
37		M	71	91	Advanced	R.LNs	Locoregional			NED	90.51^a^					90.51^b^

Abbreviations: CR, complete response; CTx, chemotherapy; D.LNs, distant lymph nodes; F, female; M, male; NE, not evaluable; NED, no evidence of disease, P.LNs, pelvic lymph nodes; PD, progressive disease; PR, partial response; R.LNs, regional lymph nodes; RT, radiation therapy; SD, stable disease.

^a^Censored data (not progressed or loss of follow‐up).

^b^Censored data (alive or loss of follow‐up).

## APPENDIX 2 Comparison of clinical characteristics between patients receiving platinum‐based chemotherapy and taxane‐based chemotherapy

**Table:**

Characteristics	Platinum‐based	Taxane‐based	*p*‐Value
Age			
≤60	10	1	0.199
>60	12	7	
Gender			
Male	17	7	1.000
Female	5	1	
ECOG PS			
0–1	12	7	1.000
≥2	3	1	
Primary sites			
Urogenital	18	7	1.000
Others	4	1	
Disease status			
Initially metastatic	8	3	1.000
Advanced/relapse	14	5	
Lymph node station			
No distant	8	3	1.000
Distant	14	5	
The sites of metastasis			
Liver/lung	7	1	0.217
Bone only	2	3	
HER2 positivity			
Positive	5	4	0.650
Negative	7	3	

Abbreviation: ECOG PS, Eastern Cooperative Oncology Group Performance Status.

## APPENDIX 3 Univariate analysis between clinical factors and objective response rate

**Table:**

Response	CR+PR	SD+PD	*p*‐Value
Age			
≤60	6	3	0.683
>60	9	8	
Gender			
Male	12	9	1.000
Female	3	2	
ECOG PS			
0–1	9	8	0.131
≥2	4	0	
Primary sites			
Urogenital	14	8	0.279
Others	1	3	
Disease status			
Initially metastatic	5	5	0.689
Advanced/relapse	10	6	
Lymph node station			
No distant	7	2	0.217
Distant	2	9	
The sites of metastasis			
Liver/lung	5	3	1.000
Bone only	4	2	
HER2 positivity			
Positive	6	4	0.620
Negative	5	2	
The type of treatment			
Platinum based	10	8	0.659
Taxane based	5	2	
CK7			
Positive	8	5	1.000
Negative	1	0	
CK20			
Positive	1	0	0.429
Negative	2	4	
CEA			
Positive	4	2	1.000
Negative	1	0	
